# Unraveling the electronic influence and nature of covalent bonding of aryl and alkyl radicals on the B_12_N_12_ nanocage cluster

**DOI:** 10.1038/s41598-023-28055-8

**Published:** 2023-01-14

**Authors:** Avni Berisha

**Affiliations:** Department of Chemistry, Faculty of Natural and Mathematics Science, University of Prishtina, 10000 Prishtina, Kosovo

**Keywords:** Materials science, Nanoscience and technology, Chemical physics

## Abstract

Carbon nanocage structures such as fullerene, nanotubes, nanocapsules, nanopolyhedra, cones, cubes, and onions have been reported since the discovery of C60, and they offer tremendous promise for investigating materials of low dimensions in an isolated environment. Boron Nitride (BN) nanomaterials such a: nanotubes, nanocapsules, nanoparticles, and clusters have been described in several studies and are predicted to be useful as electronic devices, high heat-resistance semiconductors, nanocables, insulator lubricants, and gas storage materials. The interaction, and electronic of octahedral B_12_N_12_ nanocage cluster covalently modified from the attachment of alkyl and aryl radicals were analyzed using Density Functional Theory calculations. The work discusses for the first time to our knowledge the complete investigation of the impact of the grafted aryl and alkyl groups on the electronic, bang gap, and density of states on the B_12_N_12_. Furthermore, this is the first complete description of these radicals attaching to a surface of B_12_N_12_ nanocage cluster.

## Introduction

Because of its excellent properties such as: high mechanical hardness, thermochemical stability, and electric and thermal conductivity, the boron nitride (BN) has acquired immense scientific interest^1–3^. Recently, a number of BN nanostructures, such as nanoribbons, nanomeshes, fullerenes, and hybrids with graphene have also been synthetized and used in a number of different applications (catalysis, heat transport, drug delivery^1,4–6^. The B_12_N_12_ fullerene (a 0D nanomaterial) is one of the most stable small III–V fullerenes known, with a structure made up of squares and hexagons and a network of boron nitrogen bonds that is more energetically important than those made up of pentagons and hexagons^1,7,8^. Due to its unusual chemical, physical and surface characteristics, B_12_N_12_ fullerene has been explored in recent years for biomedical^9^, drug delivery^10,11^, detection^12^ and biosensor applications^6,8,13–15^. On all of these various applications, surface modification is a critical step determining the use of material. Nanomaterials' surface modifications opens new opportunities because it affects certain properties such as dispersibility, stability, electronic and optical characteristics^16,17^. Aryl diazonium salts are commonly accepted as a gold standard to effectively modify materials in their bulk or nanoscopic form. This technique is the only overall surface modification strategy that is applied for the surface modifications of: superconducting, semi-conducting, conducting or insulating materials^18^. Despite the lack of experimental data, alkyl and aryl groups grafted onto the B_12_N_12_ nanocage cluster are explored using Density Functional Theory (DFT). Recently, DFT methods have been used to obtain molecular insights into the interaction of aryl or alkyl radicals with a range of different nanomaterials, including graphene^19,20^, graphene oxide^21^, graphyne and graphdiyne^22^, borophene^23^, 2D black phosphorus^24^, gold cluster^25,26^, and so on. DFT simulations were used to explore the diazonium-modified graphene with different functional groups from a theoretical approach. The results show that diazotization, a chemical modification of graphene, has a major influence on its properties, opening up a wide variety of possibilities in microelectronics, energy storage and conversion devices, and electrocatalysis^27,28^. The surface modification of graphene was explored, and it was shown that their binding is covalent when the coupling is done via electron deficient nitroaryl radicals formed by diazonium intermediate breakdown^29^. Berisha investigated the grafting of aryl radicals onto graphyne and graphdiyne via DFT calculations and discovered preferential binding site^22^. In this case, the calculated the Bond Dissociation Energy (BDE), which reached a value of 66 kcal/mol for the scission of the phenyl group, supports the grafted layer's remarkable stability. Furthermore, Molecular Dynamics (MD) simulation revealed that the grafted substituted aryl groups formed from aryldiazonium salts had a significant impact on the solvation characteristics of this material. DFT calculations also allowed for the correlation of spectroscopic results [obtained by surface-enhanced Raman spectroscopy (SERS)] with experimental data, demonstrating the presence of the Au-C(aryl and alkyl) bonding^25,26^.

The grafting of materials improves their usefulness and creates new potential for a varied array of applications^18,30^. The utilization of radicals formed from diazonium salts has significant benefits in this context: the salts are easily synthesized (isolated or not) from aromatic amines, many of which are commercially available, and the reaction may modify any surface, whether conductive or not. The grafting reaction can occur spontaneously or be triggered by electrochemistry, photochemistry, or other approaches. In addition, this is among a select set of compounds that establish a robust covalent bond between the surface and the aryl group, and the resulting interface is extremely stable.

The stability of the attached organic layer is important in the application of materials in areas such as sensing, photovoltaics, and so on, so evaluating this parameter, among others, is crucial. This paper investigates the geometry, binding energy, transition state, electronic properties, frontier molecular orbital, molecular electrostatic potential, and Mulliken charges of the aryl and alkyl radicals attached to the surface of B_12_N_12_ fullerene.

## Computational methods

DFT was done using the DMol3 software to speed up the computations (BIOVIA). Geometry optimization was performed inside the generalized gradient approximation (GGA-PBE)^31^ utilizing the double numerical plus polarization base set (DNP)^32^ and the Perdew-Burke-Ernzerhof functional. To account for van der Waals interactions, the Tkatchenko-Scheffler (TS)^33^ method was applied. Because there were no imaginary frequencies, the designed structures' energy minima were attained. The resultant geometry was then utilized in Gaussian. All computations in this study were done using Gaussian 16 at the B3LYP^34^/def2tzvp^34^ level of theory (with Grime's dispersion correction GD3)^35^. TDDFT, or Time-Dependent Density Functional Theory, was applied to the optimized structure in gas so that we could conduct an investigation of the UV spectra. To investigate the interaction mechanism, geometry optimization, adsorption energies, dipole moment, molecule electrostatic potential (MEP), frontier molecular orbitals (HOMO–LUMO distribution), and partial density of states (PDOS) were determined. AIMII software is used for the Quantum Theory of Atoms in Molecules (QTAIM) investigation. Other parameters such as: molecular electrostatic potential (MEP), frontier molecular orbital (FMO), partial density of states (PDOS), Electron Localization Function (ELF), Electron Density Differences Maps (EDDM) and Mulliken population analysis (MPA) as well have also been computed to gain structural details regarding the interaction of radicals with the B_12_N_12_ fullerene^4,6,14,36^.

The BDE was determined using the following formula^22,23,25^:

$${\text{BDE}} = {\text{E}}_{{{\text{B12N12}}/{\text{aryl}}\;{\text{or}}\;{\text{alkyl}}\;{\text{radical}}}} - \left[ {{\text{E}}_{{{\text{B12N12}}}} + {\text{E}}_{{{\text{Aryl}}\;{\text{or}}\;{\text{alkyl}}\;{\text{radical}}}} } \right]$$

where E_B12N12/aryl or alkyl radical_ stands for the energy of the grafted B_12_N_12_ structure. E_B12N12_ stands for the total energy of the pure B_12_N_12_ nanocage,

The wavefunction analysis and graphical representation of the derived results was performed using Multiwfn^37,38^ software and Visual Molecular Dynamics (VMD)^39^.

## Results and discussion

### Bond lengths and adsorption energies

**Figure 1:**
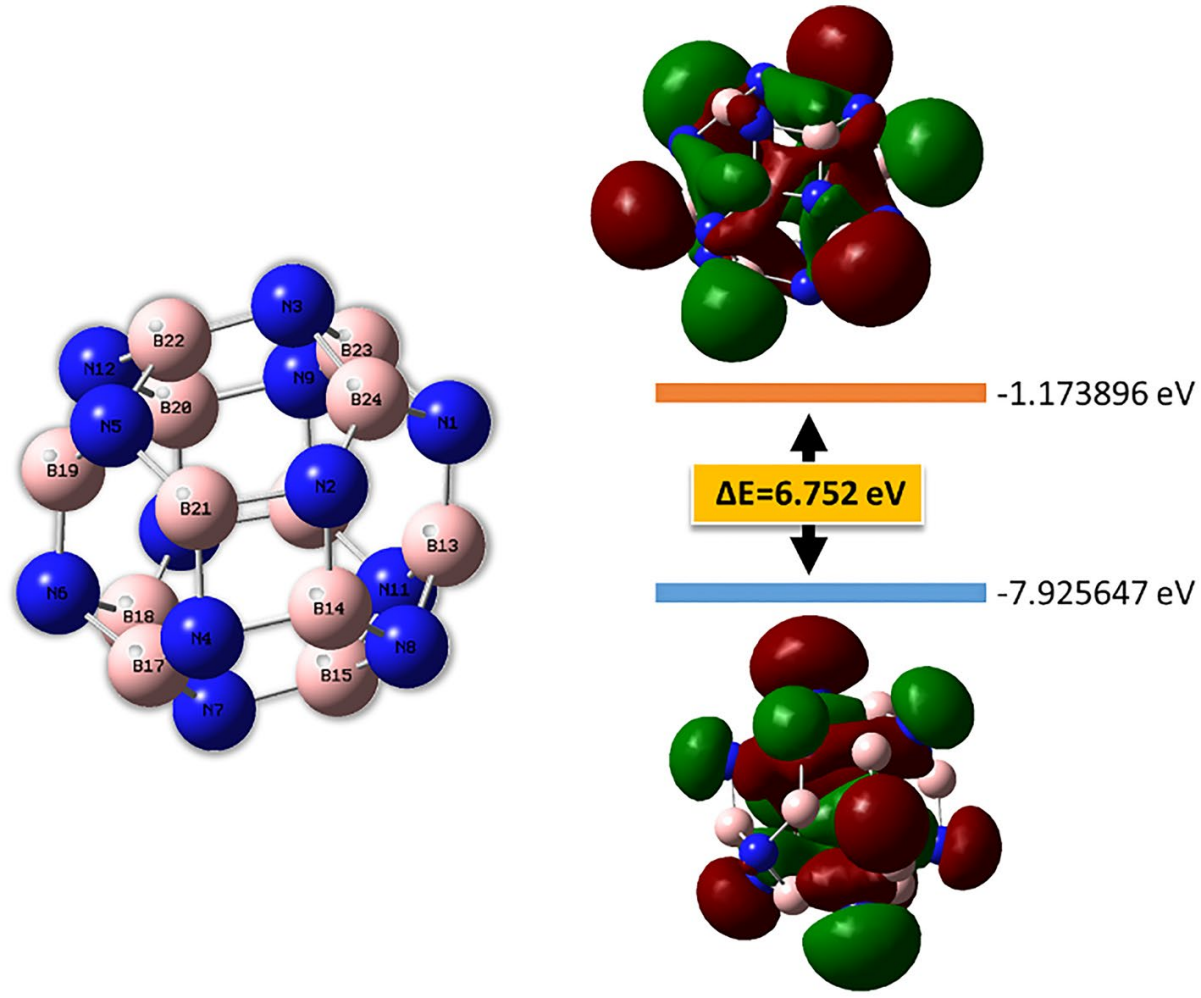
Optimized geometry and HOMO, LUMO levels of the B_12_N_12_ nanocage cluster.

Figure 1 depicts the B_12_N_12_ optimized structure at the B3LYP[/def2tzvp basis set. B_12_N_12_ structure has been optimized. In this cluster, the nitrogen and boron sites are equal. Six tetragonal (4-membered) and eight hexagonal (6-membered) rings make up the cluster. The length of the BN bond varies depending on whether the bond is between a tetragonal and a hexagonal ring (b_64_) or between two hexagonal rings or between two hexagonal rings (b_66_)^6,40^. The length of the BN bond shared by two hexagonal rings (b_66_) is 1.43783 Å, whereas the length of the BN bond shared by a tetragonal and a hexagonal ring (b_64_) is 1.48422 Å.

Grafting aryl and alkyl groups onto the BN cage, in general, induces alterations in the geometry of the BN cage. When these groups are present on the link between two hexagonal rings (atom N_2_-B_24_), the length of the BN bond increases in all cases of the grafted moieties as presents in Table 1. This is also seen to be the case for the BN bonding that exists between the tetragonal rings (atoms N_4_-B_24_). These induced structural alterations caused by radical grafting have previously been documented in gold clusters and other materials^25,41^. The bond between the grafted B24 atom of the B_12_N_12_ nanocage cluster and the C atoms of aryl or alkyl groups is close to the previously reported experimental value for B–C bond of d_(B-C)_ = 1.534 ± 0.01 Å indicating that these moieties are strongly covalently bound to the clusters surface^42^.

**Table 1:** Bond length on the selected atoms for the bare and grafted B12N12 nanocage cluster.

Bond length [Å]	B_12_N_12_	B_12_N_12_-Ph	B_12_N_12_-C_6_H_13_	B_12_N_12_-PhNO_2_
N_4_-B_24_	1.437	1.589	1.488	1.629
N_2_-B_24_	1.484	1.594	1.546	1.622
B_24_-C_aryl or alkyl_	–	1.581	1.541	1.572

We investigated the reactions that the aryl radicals (phenyl and nitrophenyl) and the alkyl radical (hexyl) had with the B_12_N_12_ nanocage cluster. It has been previously confirmed that the Bond Dissociation Energy (BDE) is a significant parameter that can be used to evaluate the strength of the interface while the grafting process is taking place^25,26,41,43–46^.

**Figure 2:**
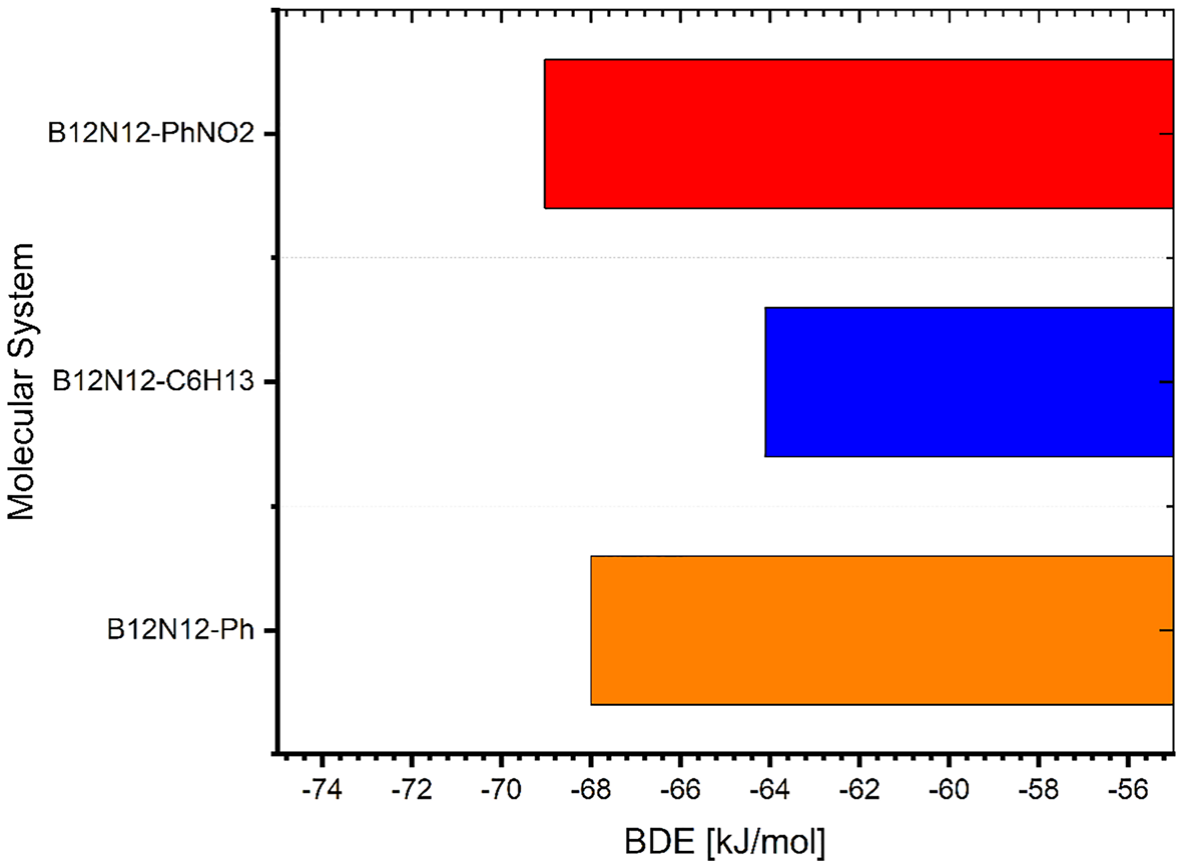
BDE values for the grafted B_12_N_12_ nanocage cluster by corresponding aryl and alkyl radical.

The calculated BDEs for the grafted groups as shown in Fig. 2 were more than 60 kcal/mol. These numbers are suggestive of the establishment of an interface that is stable^43^. The layers generated by grafting radicals are more stable than previously documented surface modification processes based on the formation of self-assembling monolayers (SAM) through thiol chemistry (BDE|Au-S-(CH_2_)_5_-COOH|= 31.59 kcal/mol).

### Dipole moment

The dipole moments of pure B_12_N_12_ and grafted B_12_N_12_ were compared and are shown in Table 2. The pure B_12_N_12_ cage has no dipole moment since it is symmetrical^40^. Binding of a–Ph, –C_6_H_12_, or PhNO_2_ group raises the dipole moment in B_12_N_12_-Ph from zero to 2.237 D, accordingly to 6.666 for –PhNO_2_, but grafting of a -alkyl group (–C_6_H_12_) results in just a minor increase of the dipole moment up to 0.882.

**Table 2:** Dipole moment of the bare and grafted B_12_N_12_ nanocage cluster.

System	B_12_N_12_	B_12_N_12_-Ph	B_12_N_12_-C_6_H_13_	B_12_N_12_-PhNO_2_
Dipole moment [Debye]	0.006	2.237	0.882	6.666

**Figure 3:**
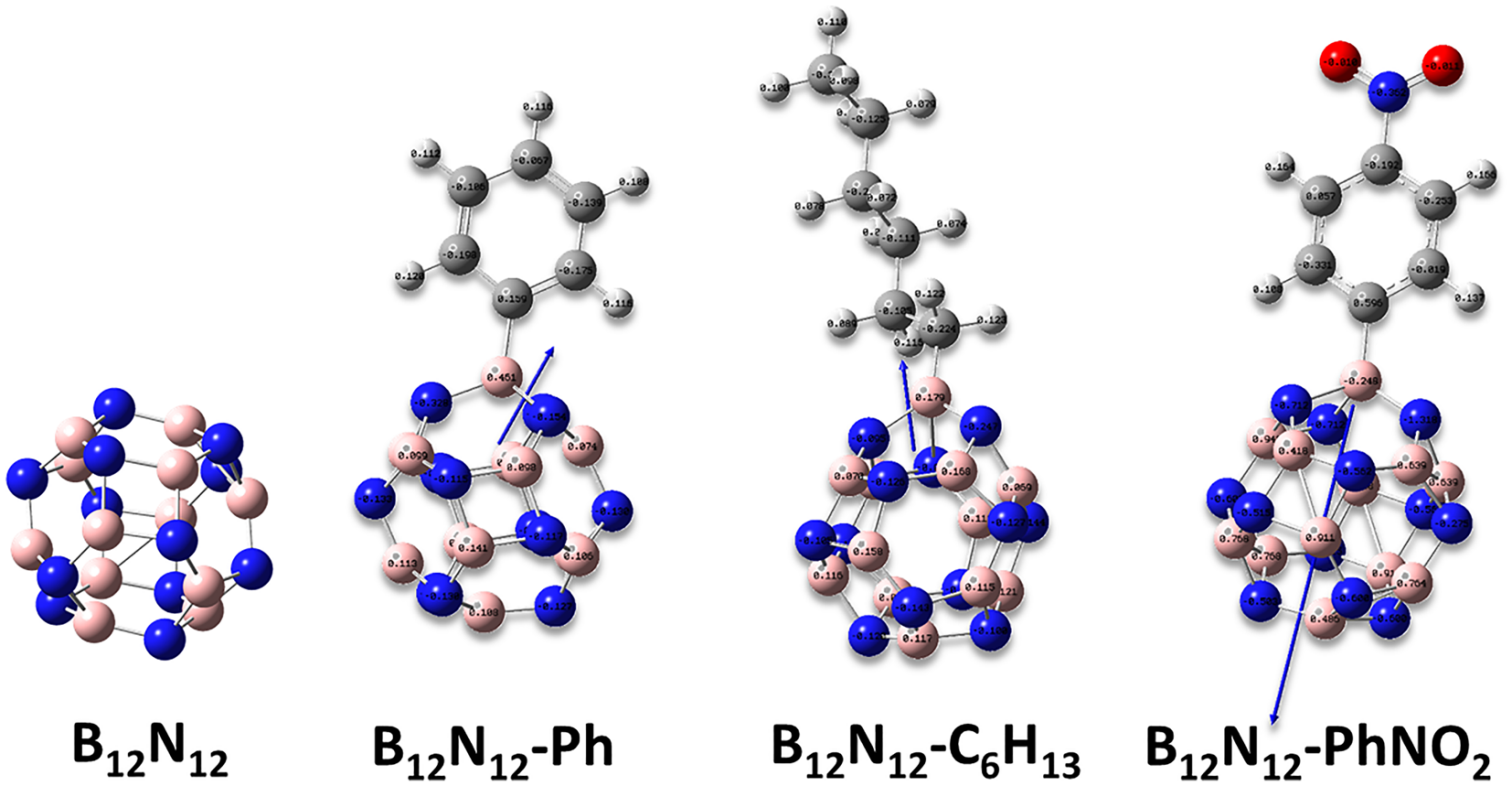
The orientation of the dipole moment of the bare and grafted B_12_N_12_ nanocage cluster.

This effect is important as not only alters the electronic properties as seen above but also it enables the dispersibility in different solvents^22^. These alterations are ascribed to aryl or alkyl group additions, which disrupt charge separation in the B12N12 nanocage. The dipole moment vectors (Fig. 2) in the grafted structures, as shown in Fig. 3, point from the grafted groups toward the B_12_N_12_ or vice versa, suggesting charge transfer from these groups to the nanocage or the inverse.

### MEP analysis

To understand the interaction between the B_12_N_12_ and the grafted aryl or alkyl group, the molecular electrostatic potential (MEP) is performed. It represents the extent of charge dispersion in a molecule and relates molecular structure to physiochemical qualities such as chemical reactivity, dipole moment, and partial charges.

**Figure 4:**
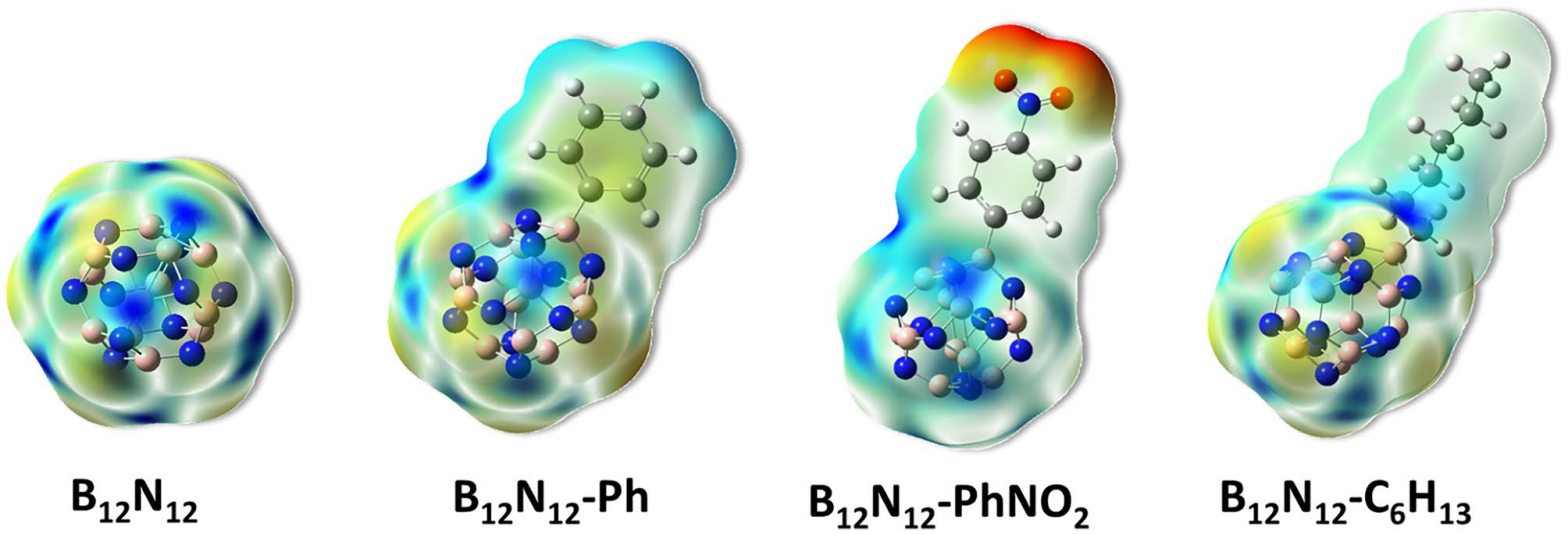
MEP surface bare and grafted B_12_N_12_ nanocage cluster.

The electron-deficient blue area (in the online version) in Fig. 4 represents boron atoms, whereas the electron-rich yellow zone represents nitrogen atoms. Because the pure B_12_N_12_ nanocage is symmetrical, it exhibits both charges to an equal amount, which alter somewhat following the grafting of alkyl or aryl groups. These groups after their grafting reduce the intensity of the blue zone on the B_12_N_12_ nanocage (shifting toward the grafted moieties).

### Electronic properties

**Figure 5:**
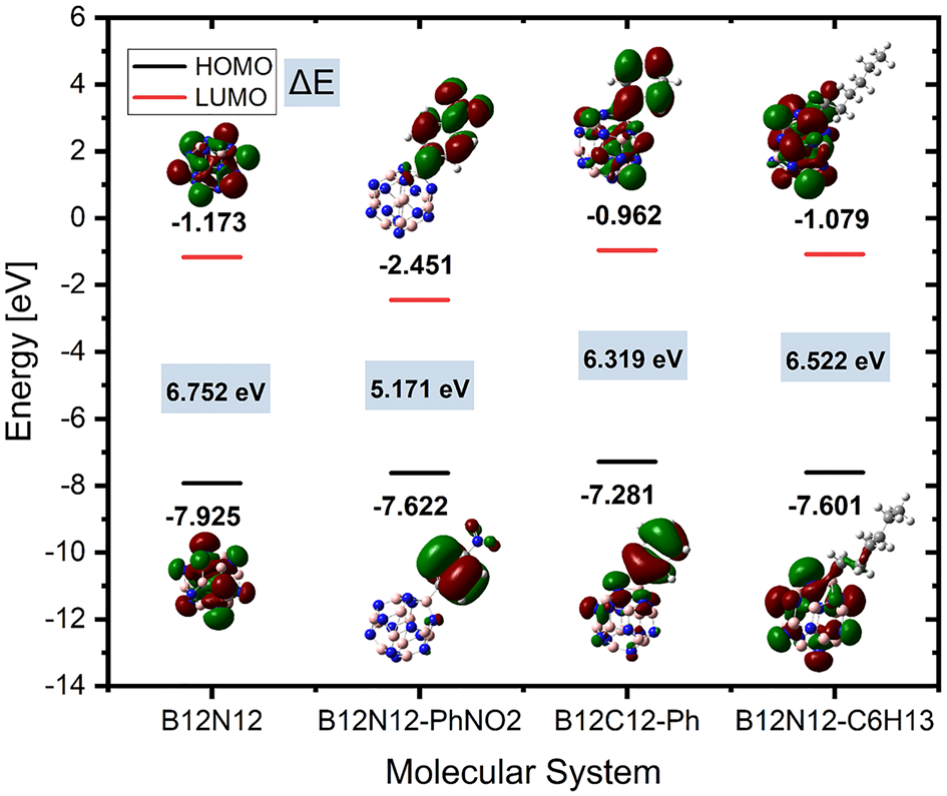
HOMO, LUMO and band gap of the bare and grafted B_12_N_12_ nanocage cluster.

The influence that the grafted groups have on the B_12_N_12_ nanocages can be seen rather well when looking at the densities as well as the electronic energy levels. Figure 5 provides details about a variety of orbital properties, including the energies of the HOMO and LUMO states as well as the HOMO–LUMO band gap (Eg). The B_12_N_12_ nanocage is a semiconductor with a HOMO LUMO gap (Eg) of 6.752 eV. The B_12_N_12_ nanocage has HOMO and LUMO values of − 7.92 and − 1.17 eV, respectively. The Fermi level, EFL, is equal to − 4.54 eV. The Fermi level denotes the center of the HOMO–LUMO energy gap (in a molecule when the temperature is 0 K). The grafting of carbon-centered radicals onto a nanocage alters the HOMO and LUMO energies and consequently the band gap of this entity. The band gap difference is reduced in all grafted instances. The HOMO–LUMO gap (Eg) is directly related to conductivity, resulting in a high energy level for the newly generated HOMO^47^. As a result of the narrowing of the energy gap between the HOMO and LUMO states, it is anticipated that there would be a significant increase in the material's electrical conductivity (Eg). This will make it possible for the resultant grafted clusters to be utilized in novel ways (electronics, photovoltaic applications, sensing, …).

The DFT results were used to calculate the band gap energy and the threshold wavelength^48^. The Tauc plot generated from DFT calculations in the gas phase and the corresponding optical band gap values can be found in the Supporting Information (Fig. S1 and Table S1)^49^. The optical bang gap as observed in many studies is often much lower than the fundamental HOMO–LUMO gap because, in the excited state (as opposed to the ionized state), the electron and hole remain electrostatically coupled to one another^50^.

### Partial density of states (PDOS)

**Figure 6:**
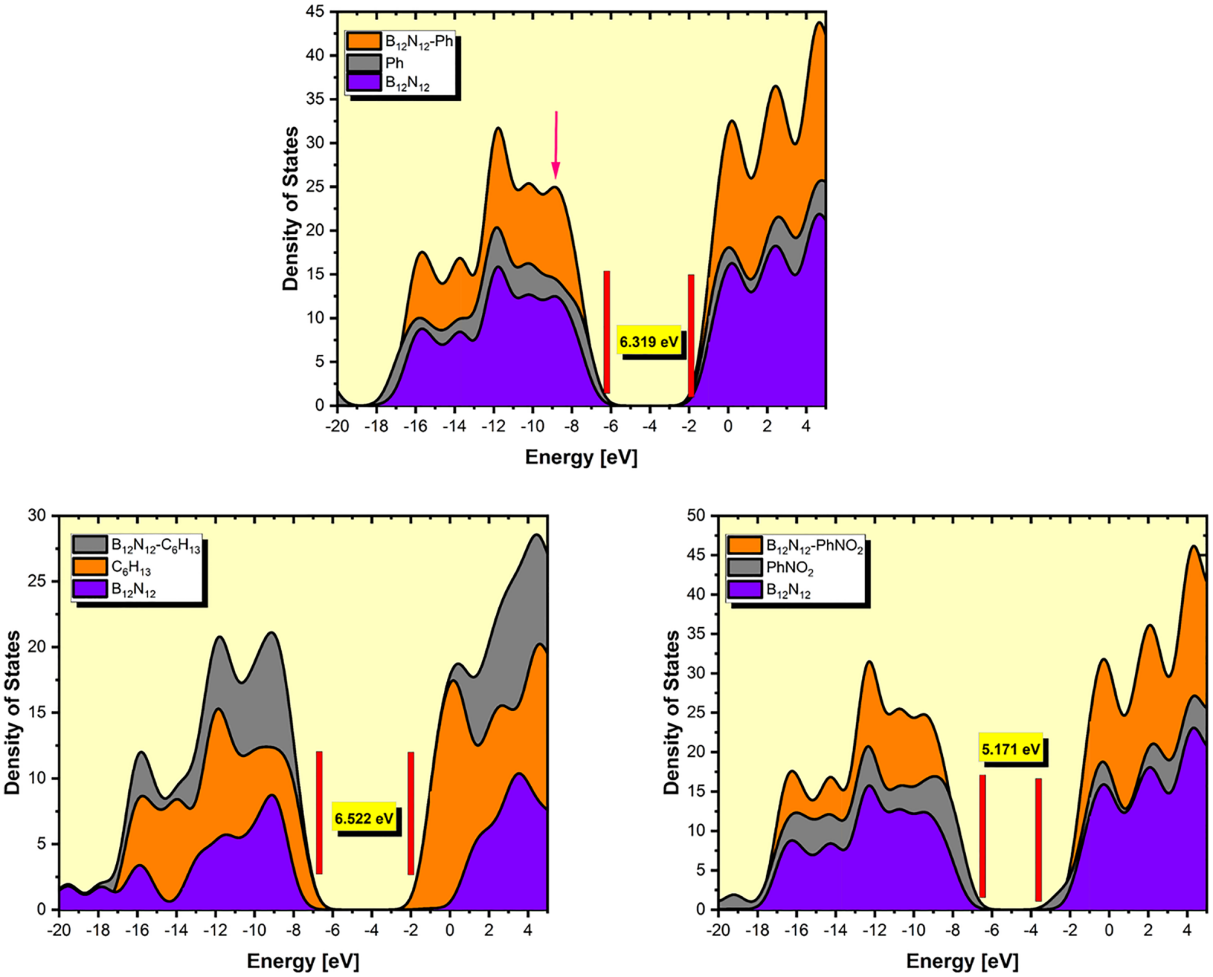
PDOS plots of the grafted B_12_N_12_ nanocage cluster.

The structural alterations and electrical characteristics of bare B_12_N_12_ and B_12_N_12_ nanocages after grafting were investigated using partial density of states (PDOS). As shown in Fig. 6, the LUMO has density primarily localized grafted groups in the case of B_12_N_12_-PhNO2, on the entire structure for B_12_N_12_-Ph, and almost on the B_12_N_12_ nanocage for B_12_N_12_-C_6_H_13_; the HOMO is centered only on the grafted phenyl groups, whereas in the case of B_12_N_12_-C_6_H_13_ it is also in the vicinity of the grafted.

### Quantum theory of atoms in molecules (QTAIM)

Electron density analysis was performed in the context of Bader's proposed quantum theory of atoms in molecules (QTAIM)^51,52^. In general, the electron density at the Bond Critical Points (BCP), ρ_(b)_, is greater than 0.20 e^−^ bohr^−3^ in shared-shell interactions, i.e., covalent bonds, and less than 0.10 e^−^ bohr^−3^ in closed-shell interactions (e.g. ionic, van der Waals, hydrogen bonding)^53,54^.

**Figure 7:**
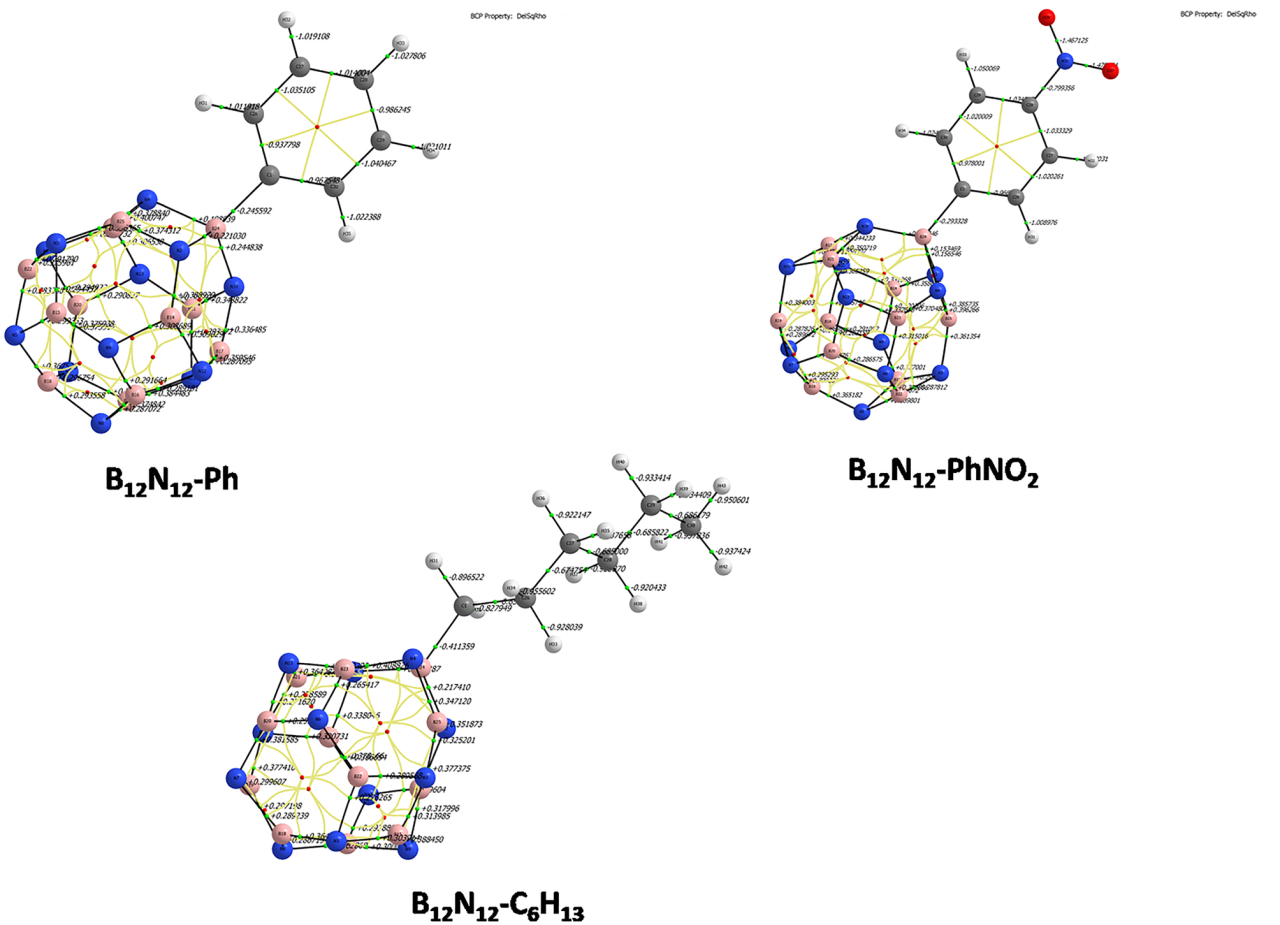
Molecular graph of the grafted B_12_N_12_ structures (the lines are bond paths).

The binding among the atoms on the grafted structures is visible in Fig. 7, by analyzing the presence of the Bond Critical Points (BCPs)—presented as green spheres. As seen in Table 3, ρ(b) is close to 0.2 e^−^ bohr^−3^, indicating that the formed B-C bond has some polarization due to differences in electronegativity among the bonded atoms [χ(C) = 2.5 and χ(B) = 1.5].

**Table 3:** Obtained valiues of QTAIM parameters of the bare and grafted B_12_N_12_ nanocage cluster.

Molecular system	ρ(b) [bohr − ^3^]	∇^2^ρ(b)	V(b)	G(b)	|Vb|/Gb	|Vb|/(2 Gb)	Delocalization index δ(Ω,Λ)
B_12_N_12_-PhNO_2_	0.191	− 0.2933	− 0.3570	0.1418	2.517	1.25	0.462
B_12_N_12_-Ph	0.185	− 0.2456	− 0.3490	0.1438	2.427	1.23	0.436
B_12_N_12_-C_6_H_13_	0.205	− 0.4113	− 0.3787	0.138	2.744	1.372	0.515

Another energetic descriptor that is frequently used to distinguish two types of closed-shell bonding is the |Vb|/Gb ratio, which reflects the covalency magnitude of the interaction. If the latter ratio is less than one, the kinetic energy density is the leading term, and electrons are destabilized near the BCP, implying that no covalency is expected (for example pure ionic or van der Waals bonding). These interactions are referred to as pure closed-shell interactions (pure CS). The second type of closed-shell bonding involves some electron sharing (|Vb|/Gb > 1, indicating that the potential energy density is high and electrons are stabilized at the BCP)^54,55^. In the case of B_12_N_12_ grafted cluster the |Vb|/Gb is > 1 indicating that there is a close shell type of bonding with electron some sharing. The delocalization index, DI, or δ(Ω,Λ) is a quantitative tool used to assess the extent of electron sharing in the context of QTAIM. DI is close to zero for an ideal ionic system, but close to unity for homo-nuclear covalently bonded systems, two for double bonds, and so on^56^. It is a direct measure of electron sharing that reflects covalency. This supports the fact that the B-C bond has a covalent-polarized character.

### ELF

In order to gain a better understanding of the nature of the new covalent chemical bonds that are forming between the carbon atom on the alkyl or aryl radical and the boron atom that is a part of the B_12_N_12_ nanocage cluster, we have further calculated the Electron Localization Function (ELF)^23,57^. By establishing a renormalization of the Fermi hole curvature, the ELF serves as a measure for electron pairing (localization). Values for the ELF range from zero (no electron localization) to one, with zero indicating that there is no electron localization and one indicating that there is complete electron localization (electron pairing, covalent bond).

**Figure 8:**
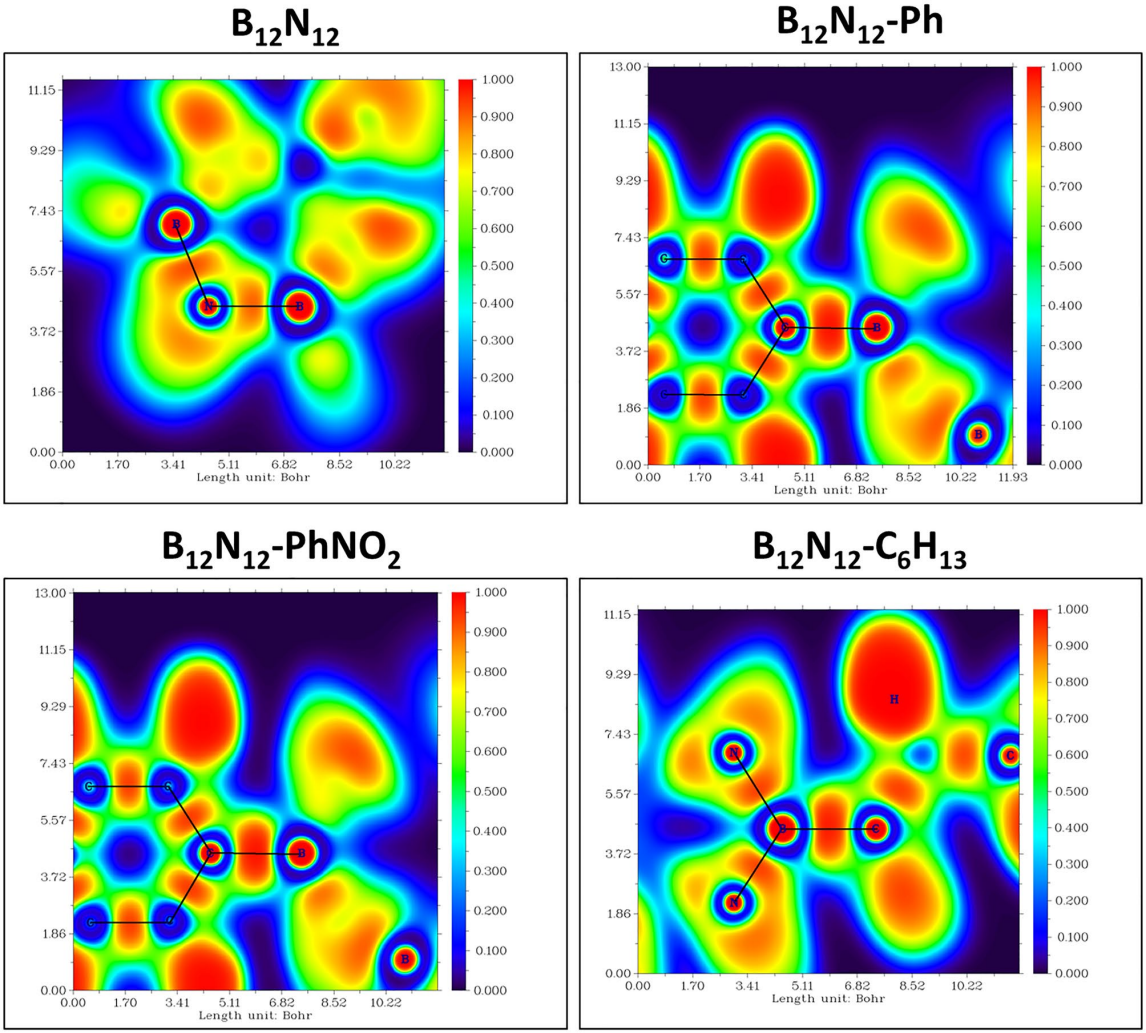
ELF plot of the binding B atom of the B_12_N_12_ nanocage and the C atom of the corresponding aryl or alkyl radical.

Covalent bonding may be recognized in Fig. 8 as the maxima of ELF occurring along the bond almost halfway between the two atoms (B-C). It is evident from the ELF depicted in Figure that the bonding between the two atoms in question is of the covalent type. As seen in this figure, the presence of a red region in the center of two carbon atoms demonstrates that the B–N bond on the nanocluster is actually of the covalent nature. These results are supported also through the analysis of the bond order: Mayer Bond order^58,59^, Fuzzy Bond Order (FBO)^60^ and Laplacian Bond Order (LBO)^61^ between the bonded atoms as presented in Table 4.

**Table 4:** Bond orders for the B–C atoms of the grafted clusters.

Bond order	B_12_N_12_-Ph	B_12_N_12_-C_6_H_13_	B_12_N_12_-PhNO_2_
Mayer bond order	0.907	1.101	1.101
Fuzzy bond order (FBO)	0.903	1.065	1.065
Laplacian bond order (LBO)	1.034	1.187	1.077

The electron density is split in such a way by the Mayer bond order that the degree of bonding can be determined in a straightforward manner. According to this order, the value assigned to a completely fulfilled double bond is 2, the value assigned to a triple bond is 3, and so on^62^. The bond order values are rather near to one, showing once more the presence of covalent single bonds between the B-C atoms of the grafted moieties, as opposed to multiple bonds. In order to be independent of the calculation methods (the usage of basis set), the FBO were also computed; typically, the magnitude of the FBO is similar to the Mayer bond order, particularly for low-polar bonds, but is considerably more stable with regard to the change in basis set. LBO presented a new concept of covalent bond order based on the Laplacian of electron density ∇^2^ρ in fuzzy overlap space^61^. LBO was shown to be logical and helpful by applying it to a wide range of compounds and comparing it to various current bond order classifications. It is demonstrated that LBO has a direct relationship with bond polarity, bond dissociation energy, and bond vibrational frequency. LBO has a low computational cost and is indifferent to the computing level utilized to create electron density. The numbers corroborate the atom bonding order; the calculated values are quite near to Mayer bond orders.

### Electron density difference (EDD) analysis

When a chemical bond is formed, an important rearrangement of the electrons in the system takes place, which results in polarization and the transfer of charge. In particular, the creation of a covalent bond must be accompanied by the phenomena of electrons congregating in the bonding area. This may be shown by plotting an electron density difference (EDD) plot, which is one of the most effective ways to do so.

**Figure 9:**
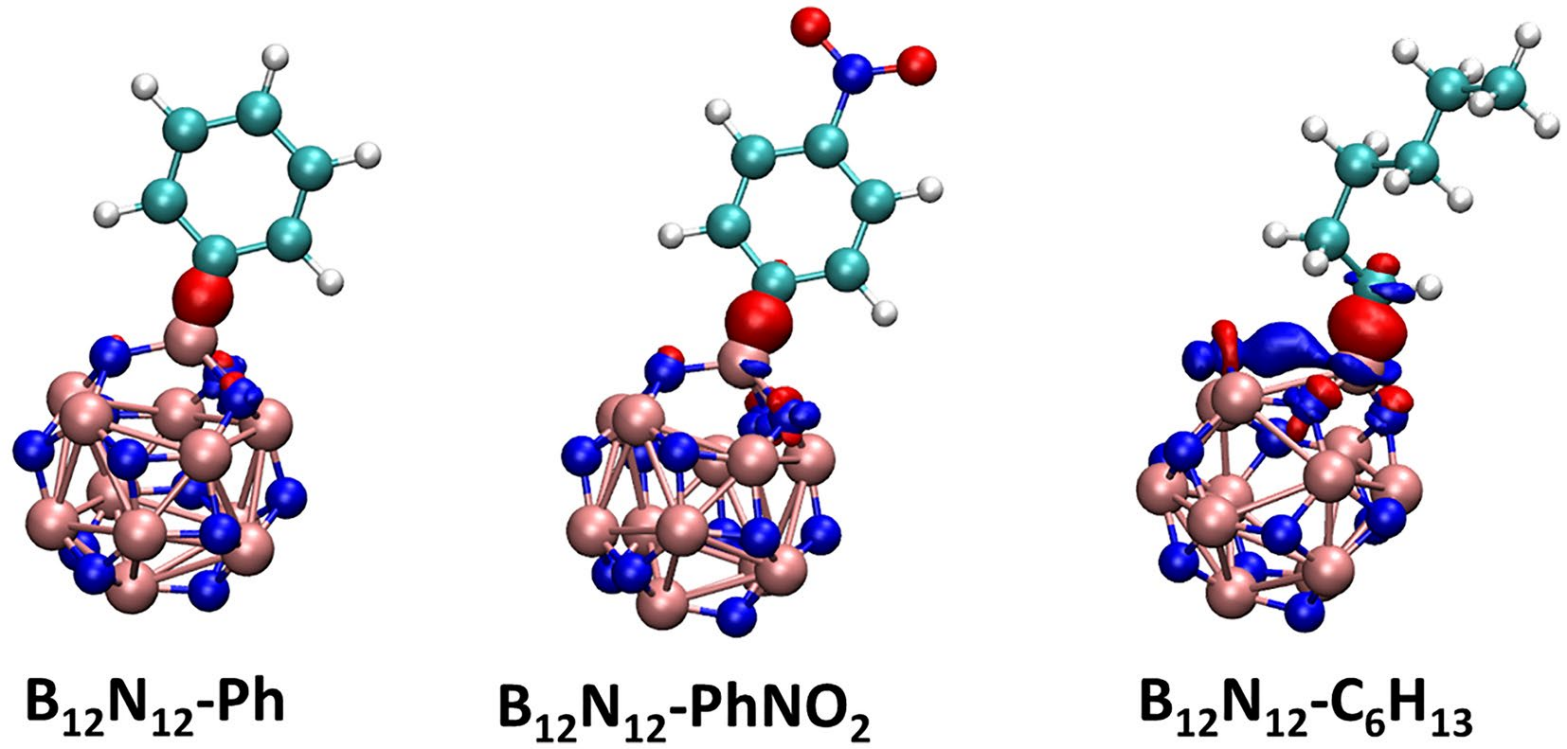
EDD for the grafted B_12_N_12_ clusters.

In the Fig. 9, we can observe that the interaction includes charge transfer mostly between the C atom and the surrounding B atoms in B_12_N_12_ by referring to the charge density difference diagrams of aryl or alkyl groups and B_12_N_12_ cluster. EDD show that there is a concentration of electron density (red color in map) between C and B atom supporting the bond formation. Additionally, the formation of a chemical connection between two distinct fragments must result in measurable charge transfer (CT). CT may be calculated as the difference between the fragment charge in the actual system and the net charge of the fragment in its isolated condition. The fragment charge is defined as the total of the charges of the fragment's atoms^37^. It's worth noting that the amount of charge regarding the charge fragments of the various bound groups differs, for:–C_6_H_13_ (q = − 0.0237),–Ph (q = 0.091) and –PhNO_2_ (q = − 0.151)—this is probably what influences the BDE values and the Bond orders for the B-C atoms.

## Conclusion

We used the DFT calculations to investigate the grafting of aryl and alkyl radicals on B_12_N_12_ nanocages in this work. The computed BDEs for the grafted groups were greater than 60 kcal/mol, indicating the formation of a stable interface. After grafting, B_12_N_12_ exhibits significant changes in its electronic properties. The dipole moment of the grafted system increases as well. The HOMO/LUMO gap in bare B_12_N_12_ is greater than in other grafted systems. Furthermore, the partial density of states and electronic energy level were computed to demonstrate the influence of the grafted moieties on the structure of B_12_N_12_. The ELF, QTAIM, EDD, CT and bond order all clearly demonstrate that the generated bond between the B atom of the B_12_N_12_ and the grafted aryl or alkyl groups is polarized covalent.

## Supplementary Information

Supplementary Information.

## Data Availability

The datasets generated during and/or analysed during the current study are available from the corresponding author on reasonable request.
