# Supplementary material for: Unraveling the electronic influence and nature of covalent bonding of aryl and alkyl radicals on the B12N12 nanocage cluster
Source: Sci Rep. 2023 Jan 14;13:752. doi: 10.1038/s41598-023-28055-8 (PMC9840632; doi:10.1038/s41598-023-28055-8)
Supplement: Supplementary file 1 — Supplementary Information. [file 41598_2023_28055_MOESM1_ESM.docx]

**Unraveling the electronic influence and nature of covalent bonding of aryl and alkyl radicals on the B_12_N_12_ nanocage cluster**

**Avni Berisha ***

Department of Chemistry, Faculty of Natural and Mathematics Science, University of Prishtina, 10000 Prishtina, Kosovo (email: [avni.berisha@uni-pr.edu](mailto:avni.berisha@uni-pr.edu); orcid.org/0000-0002-3876-1345)

**Supporting Information**


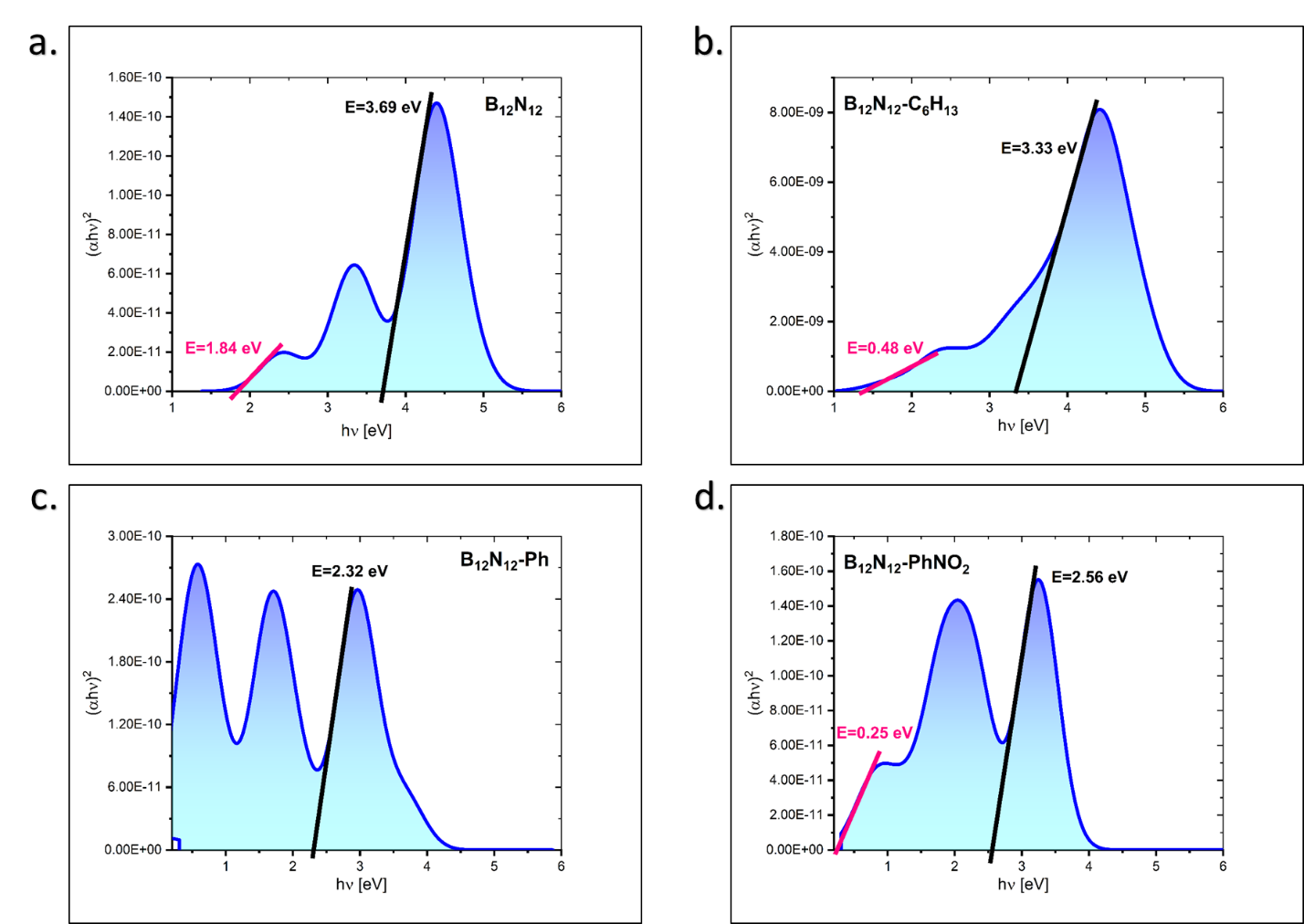


**Figure S1.** Determination of the optical band gap of bare and grafted B_12_N_12_ nanocage cluster.

**Table S1.** Band gap and threshold wavelengths value for the bare and grafted B_12_N_12_ nanocage cluster.

| **Molecular system** | **E_g_ [ev]** | |
| --- | --- | --- |
|  | **(First peak)** | **(Second peak)** |
| **B_12_N_12_** | 3.69 | 1.84 |
| **B_12_N_12_-PhNO_2_** | 3.33 | 0.48 |
| **B_12_N_12_-Ph** | 2.32 | 0 |
| **B_12_N_12_-C_6_H_13_** | 2.56 | 0.25 |
